# ORF45-induced Filamin A phosphorylation promotes cell motility and cell-contact dependent viral infection of Kaposi’s sarcoma-associated herpesvirus

**DOI:** 10.1371/journal.ppat.1013737

**Published:** 2025-11-24

**Authors:** Xiaojuan Li, Li Quan, Rihong Zhou, Xiangpeng Liu, Ronit Sarid, Ersheng Kuang

**Affiliations:** 1 Institute of Human Virology, Zhongshan School of Medicine, Sun Yat-Sen University, Guangzhou, Guangdong, China; 2 College of Clinical Medicine, Hubei University of Chinese Medicine, Wuhan, Hubei, China; 3 Hubei Shizhen Laboratory, Wuhan, Hubei China; 4 The Mina and Everard Goodman Faculty of Life Sciences & Advanced Materials and Nanotechnology Institute, Bar-Ilan University, Ramat Gan, Israel,; 5 Key Laboratory of Tropical Disease Control (Sun Yat-Sen University), Ministry of Education, Guangzhou, Guangdong, China; UPMC Hillman Cancer Center, UNITED STATES OF AMERICA

## Abstract

Kaposi’s sarcoma-associated herpesvirus (KSHV) is the etiological agent of Kaposi’s sarcoma, primary effusion lymphoma and multicentric Castleman disease. Studies have shown that cell-to-cell viral infection plays a key role in KSHV transmission in vivo, and differentiated B cells and endothelial cells might represent two distinct kinds of natural donors or recipients that radically support the lytic cycle of KSHV. Consistent with the observation that endothelial cells exhibit better acceptance and transmissibility than B lymphocytes in cell-cell contact-mediated KSHV transmission, the sequential cell detachment, migration and cell-cell contact is the determinant for this kind of viral transmission. To investigate the processes and regulation of cell-cell contact-mediated viral infection during KSHV lytic replication, we found that Filamin A, a key regulator of cell adhesion and motility, is phosphorylated in KSHV-infected adherent cells by lytic replication and ORF45 expression in an RSK-dependent manner. ORF45-induced Filamin A phosphorylation is important for cell detachment and migration, while both Filamin A knockout and S2152A knockin abolish this function. Interestingly, ORF45 deficiency, Filamin A knockout and S2152A knockin dramatically decreases KSHV de novo infection and cell-contact dependent viral infection in adherent cells. Taken together, our results demonstrated that the ORF45-Filamin A phosphorylation axis promotes cell detachment and migration and facilitates viral de novo infection and cell-to-cell transmission during KSHV lytic cycles.

## Author summary

The adherent cells undergoing KSHV lytic replication gradually become round and detached during the lytic cycle; however, how and why this phenotype occurs during KSHV lytic replication remain unknown. Alternatively, the adhesive property and cell motility of adherent cells under lytic replication are unclear, especially those of endothelial cells and keratinocytes that radically support the KSHV lytic cycle for viral transmission and tumorigenesis. Here, we reveal that ORF45 induced Filamin A phosphorylation through RSK activation during KSHV lytic cycle, and then, the cells undergoing lytic replication exhibited increased cell detachment and migration. Subsequently, ORF45-induced Filamin A phosphorylation facilitated KSHV de novo infection and cell-contact mediated viral infection. These results indicate that ORF45 facilitates cell motility of KSHV-infected cells undergoing lytic replication and promotes cell-to-cell viral transmission though Filamin A phosphorylation.

## Introduction

As an oncogenic γ2-herpesvirus, Kaposi’s sarcoma-associated herpesvirus (KSHV) is etiologically associated with three kinds of malignancies in AIDS and other immunosuppressed patients: Kaposi’s sarcoma, primary effusion lymphoma and multicentric Castleman disease [1–3]. KSHV infection has two different cycles, only a few viral genes are expressed without production of infectious viral particles in latent cycle, while all viral genes are expressed and viral DNA is replicated and encapsidated into infectious virions during the lytic cycle. In KS lesions, the majority of spindle cells are latently infected, but spontaneous KSHV lytic replication occurs in a small percentage of cells [4]. Unlike other tumor viruses, KSHV latency alone is not sufficient to induce tumors and KSHV-infected endothelial cells tend to lose latent genomes; thus, recurrent lytic replication and reinfection are needed for the virus to persistently infect KS lesions [5].

Although most human cells can be latently infected by KSHV in vitro, only lymphatic endothelial cells, terminally differentiated B lymphocytes and keratinocytes support the natural KSHV lytic cycle [6–8]. Due to the low yield of virions and the low efficiency of viral infection, cell-free viral particles are difficult to transmit and spread viral infection in natural culture, indicating that cell-free viral particles are not the main mode for viral transmission and spread. Studies have shown that cell-to-cell viral infection plays a key role in KSHV transmission [9,10], and endothelial cells and B cells might represent two distinct kinds of natural donors or recipients of KSHV infection in vivo.

ORF45 is a highly expressed viral phosphoprotein from the immediate-early stage through the late stage of the lytic cycle and is eventually incorporated into virion particles as a tegument protein [11,12]. ORF45-null mutagenesis substantially attenuated viral lytic replication, virion production and primary infection, suggesting that ORF45 plays important roles in both the initial and late stages of viral infection [13,14]. ORF45 is a multifunctional protein that interacts with several cellular proteins to hijack cellular pathways for lytic replication. ORF45 efficiently inhibits the expression of type I interferon genes by interacting with interferon regulatory factor 7 (IRF-7) [15] and regulates the intracellular transport of newly formed viral particles through interaction with the kinesin-2 motor protein KIF3A [16]. This protein also interacts with p90 ribosomal S6 kinase (RSK) and contributes to sustained ERK-RSK activation, which plays essential roles during lytic replication [17,18]. In addition, Siah-1/2 interacts with ORF45 to mediate its ubiquitination and degradation through the proteasome [19]. Notably, the proteasomal degradation of ORF45 is also associated with the immediate-early KSHV protein RTA [20]. Mono-ubiquitination of ORF45 facilitates maturation of budding virions in the trans-Golgi and endosomes through an unknown mechanism [21]. The recent studies also reveal that ORF45 up-regulates ATF4-LAMP3 expression and directly interacts with FoxK1/K2, to promote the lytic gene expression during late KSHV lytic replication [22–24].

Although knowledge of ORF45 during KSHV infection and diseases is still limited, the function of ORF45-RSK signaling has been identified through different approaches. Similar to the ORF45-null virus, a mutated virus encoding ORF45-F66A, which fails to interact with and activate RSK, exhibits reduced late lytic viral gene expression, a 5–10-fold decrease in production of infectious progeny viruses and reduced infectivity in de novo infection compared to wild-type viruses [14]. Alternatively, an inhibitory peptide that disrupts the ORF45-RSK interaction has been developed to inhibit spontaneous and chemical-induced KSHV lytic replication [25], providing a promising peptide agent for controlling KSHV lytic infection. Mechanistically, ORF45-mediated RSK activation promotes transcription and translation through the induction of c-Fos and eIF4B phosphorylation during the lytic cycle [26,27]. Importantly, a phosphoproteomic analysis identified the phosphorylated proteins during KSHV lytic replication as well as the cellular substrates of RSK induced by ORF45 [28], suggesting that ORF45-RSK signaling regulates diverse cellular and viral activity and behaviors.

Filamin A is an actin-binding protein that connects adjacent actin filaments and links actin filaments to membrane glycoproteins to regulate cell shape, attachment and migration by remodeling the cytoskeleton [29,30]. As a scaffold protein, it interacts with many intracellular proteins, including integrin, transmembrane proteins, and signaling molecules, to affect the intracellular trafficking, activity, morphology and behavior of organelles [31–33]. The functions of Filamin A are regulated by diverse kinases; phosphorylation of Filamin A at Serine 2152 is mediated by RSK and other kinases to control cell activity and behavior [34–39], whereas phosphorylation of Filamin A by CDK1 at other sites is important for successful cell division [40].

In the present study, we reveal that Filamin A phosphorylation is induced by ORF45-mediated RSK activation during KSHV primary infection and lytic replication; consequently, ORF45-induced Filamin A phosphorylation promotes the detachment and migration of lytic KSHV-infected cells and facilitates KSHV de novo infection and cell-to-cell viral transmission through cell contact and movement during the lytic cycle.

## Results

### ORF45 induces Filamin A phosphorylation during KSHV lytic replication

**Fig 1:**
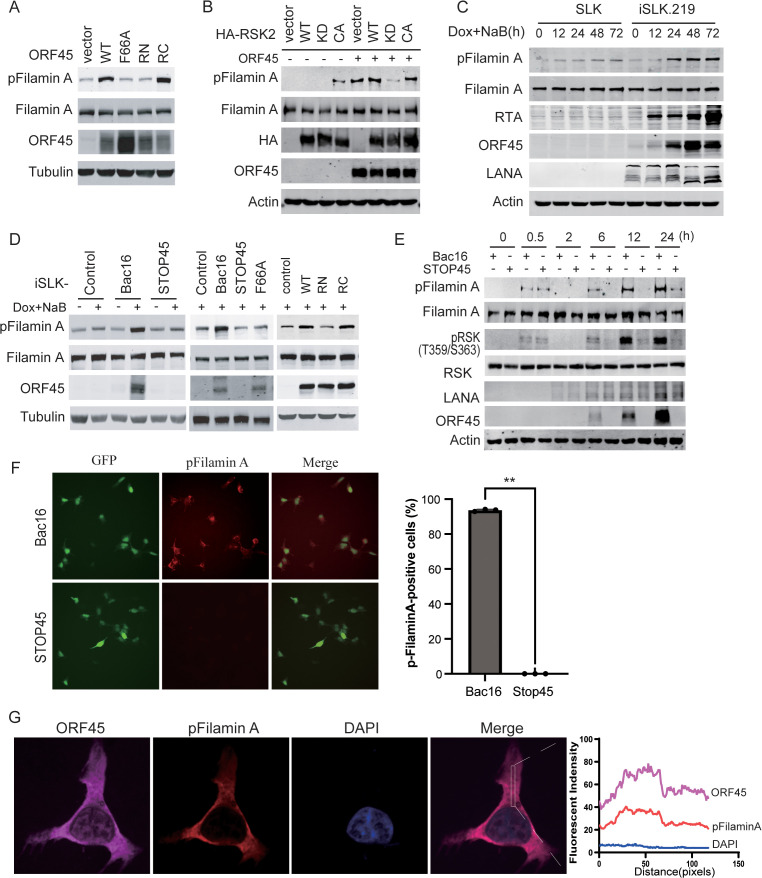
Filamin A phosphorylation is induced by ORF45-mediated RSK activation. A. Ectopic ORF45 WT and F66A, RN or RC mutated constructs were introduced into SLK cells by infection with recombinant lentiviruses. The cells were collected at 72 h post-infection, and whole cell lysates were analyzed by Western Blots as indicated. B. HEK293 cells were cotransfected with HA-RSK2 WT-, CA- or KD-expressing plasmids with empty vector or ORF45-expressing plasmids for 24 h, and then, the cells were serum-starved overnight, collected and subjected to western blot analysis as indicated. C. SLK cells and iSLK.219 cells were induced with 1 µg/ml Dox and 1 mM NaB. The cells were collected at different time points, and whole cell extracts were detected as indicated. D. Control cells, iSLK-Bac16, -STOP45, ORF45F66A, ORF45RN or ORF45RC cells were induced with Dox and NaB as described above, the cells were collected at 72 h after induction, and whole cell extracts were analyzed. E. HEK293 cells were infected with Bac16 or STOP45 virions (MOI = 10). At the different time points post-infection, the cells were collected and whole cell extracts were prepared and subjected to Western Blotting analysis as indicated. F. The iSLK.Bac16 or iSLK.STOP45 cells were induced with Dox and NaB for 48 h, and then fixed and stained with anti-pFilamin A antibody with Alexa-555 secondary antibody, and finally visualized with confocal fluorescence microscopy. The percentages of pFilamin A-positive cells in cells undergoing lytic reactivation were calculated in three independent experiments and shown. **, p < 0.01; t test. G. The iSLK.Bac16 cells were induced with Dox and NaB for 48 h, and then fixed and stained with anti-ORF45 antibody with Alexa-647 secondary antibody and anti-pFLNA antibody with Alexa-555 secondary antibody, and finally visualized with confocal fluorescence microscopy. The subcellular co-localization of ORF45 and pFilamin A was analyzed and shown.

To further investigate the novel function of ORF45-mediated RSK activation, we detected the phosphorylation of Filamin A in the ORF45-expressing cells and found that wild-type ORF45 (WT), but not ORF45-F66A, induced Filamin A phosphorylation (Fig 1A). We identified the nuclear export signal and nuclear localization signal in ORF45 and constructed ORF45 mutants that are restricted exclusively to either the cytoplasm or the nucleus [41]. The nucleus-residing ORF45 (RN) lost this ability to induce Filamin A phosphorylation while cytoplasm-residing ORF45 (RC) maintained its full function in Filamin A phosphorylation (Fig 1A). To confirm the role of RSK in ORF45-induced Filamin A phosphorylation, we coexpressed the different RSK2 constructs with ORF45, and we found that the constitutively active RSK2 (CA) construct alone induced Filamin A phosphorylation, similar to ORF45 alone. ORF45 and RSK2 WT or CA coexpression augmented phosphorylation, while the kinase-dead RSK2 (KD) construct attenuated it (Fig 1B). Furthermore, the treatment with the RSK inhibitor BI-D1870 or SL0101 dramatically attenuated Filamin A phosphorylation in ORF45-expressing cells (S1A Fig). These results suggest that ORF45 induces Filamin A phosphorylation in an RSK-dependent manner.

Furthermore, we detected Filamin A phosphorylation during KSHV lytic replication and found that Filamin A phosphorylation was increased following ORF45 expression in the iSLK.219 cells under doxycycline (Dox) and NaB induction, while no change was observed in the uninfected SLK cells under the same conditions (Fig 1C). During lytic reactivation, the level of Filamin A phosphorylation was greatly increased in wild-type Bac16-harboring cells but not in ORF45-null Bac16-Stop45-harboring cells (Fig 1D, left panel). Similarly, Filamin A phosphorylation was minimally increased in Bac16-ORF45F66A-infected cells under conditions of lytic induction (Fig 1D, middle panel). Viruses that primarily express nucleus-restricting (RN) ORF45 retain the ability to produce progeny viruses similar to viruses that express wild-type ORF45, whereas viruses expressing cytoplasm-residing (RC) ORF45 lose this ability similar to the ORF45-null virus [41], where Filamin A phosphorylation was induced by cytoplasm-residing ORF45 but not by nucleus-restricting ORF45 (Fig 1D, right panel). These results indicated that increased Filamin A phosphorylation requires ORF45 localization in the cytoplasm, whereas nucleus-localized ORF45 loses the ability to induce Filamin A phosphorylation. When Bac16 cells undergoing lytic replication were treated with the RSK inhibitor BI-D1870 or SL0101, the increased level of Filamin A phosphorylation was abolished (S1B Fig), suggesting that RSK activation is essential for Filamin A phosphorylation during lytic reactivation. Furthermore, the timing of Filamin A phosphorylation was investigated during KSHV primary infection, the level of Filamin A phosphorylation was immediately induced by both Bac16 and STOP45 viruses at 30 min post-infection probably through virion-binding cell surface receptors. Filamin A phosphorylation disappeared at 2 h post-infection and then the level strongly increased again starting from the immediately early stage after infection with Bac16 viruses (6 h post-infection) whereas infection with STOP45 viruses weakly increased the level at the delayed early stage (24 h post-infection), This finding is consistent with the timing of RSK phosphorylation and ORF45 expression (Fig 1E). The increased level of Filamin A phosphorylation during primary infection with Bac16 viruses was also abolished when cells were treated with the RSK inhibitor BI-D1870 or SL0101 (S1C Fig). These results suggest that KSHV primary infection induces Filamin A phosphorylation at early stage mainly through ORF45-induced RSK activation. When iSLK.Bac16 and iSLK.STOP45 were induced by Dox and NaB treatment and then the Filamin A phosphorylation was detected by immunofluorescence staining, a high level of Filamin A phosphorylation was observed in iSLK.Bac16 cells but not in iSLK.STOP45 cells under Dox-NaB induction (Fig 1F). The phosphorylated Filamin A mainly co-localized with ORF45 in cytoplasmic compartment of iSLK.Bac16 cells undergoing lytic reactivation (Fig 1G). These results suggest that ORF45 induces cytoplasmic Filamin A phosphorylation in cells undergoing lytic replication. Thus, we concluded that ORF45 induces Filamin A phosphorylation during lytic replication through ORF45-induced RSK activation.

### ORF45 enhances cell detachment and migration

**Fig 2:**
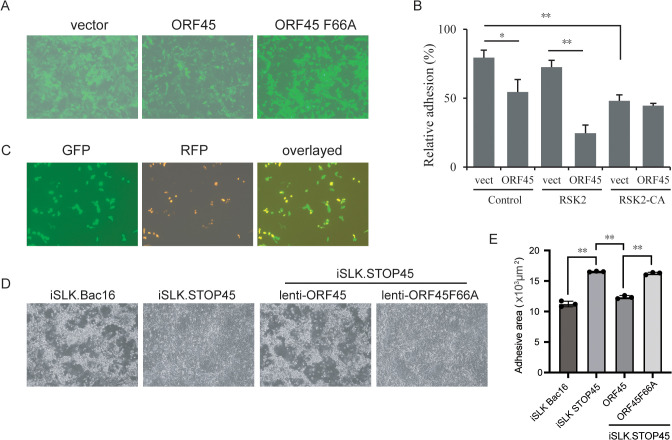
ORF45 reduces cell adhesion through RSK activation. A. HEK293 cells were infected with IRES-zsGreen empty vector, ORF45 WT or F66A-expressing lentiviruses (MOI = 2). Forty-eight hours later, the cells were dissociated and seeded on a fibronectin-coated 96-well plate. After 1 h incubation, the unattached cells were removed by low shear washing, the attached cells were observed and recorded under fluorescence microscopy, and a representative image was shown. B. HEK293 cells transfected with ORF45 and RSK2 WT- or mutant-expressing plasmids were dissociated and seeded on a fibronectin-coated 96-well plate, and the relative focal adhesion was calculated and shown as mean ± standard deviation (SD). *, p < 0.05; **, p < 0.01; t test. C. Cell detachment is induced during lytic replication. iSLK.219 cells were seeded on SLK cell monolayers at a 1:50 ratio and then induced with Dox and NaB for 48 h. The living cells were fixed and directly visualized with confocal fluorescence microscopy. D. iSLK-Bac16 cells and iSLK-STOP45 cells were induced with Dox and NaB, or iSLK.STOP45 cells were infected with ORF45 WT or F66A-expressing lentiviruses (MOI = 5) and then cells were induced with Dox and NaB at 12 h post-infection. The cells were visualized with inverted microscopy at 72 h post-induction. The quantification of relative cell adhesion was calculated and shown. **, p < 0.01; t test.

Since Filamin A phosphorylation regulates cell adhesion and migration [42,43], we examined whether ORF45 affects cell morphology and adhesion. In HEK293 cells, ORF45 overexpression reduced cell adhesion (Fig 2A and 2B), whereas this reduction was not observed when ORF45-F66A was expressed (Fig 2A). ORF45-mediated reduction was augmented by RSK2 overexpression, and constitutively active RSK2 induced cell detachment regardless of ORF45 expression (Fig 2B), suggesting that ORF45-induced RSK activation reduces cell adhesion.

**Fig 3:**
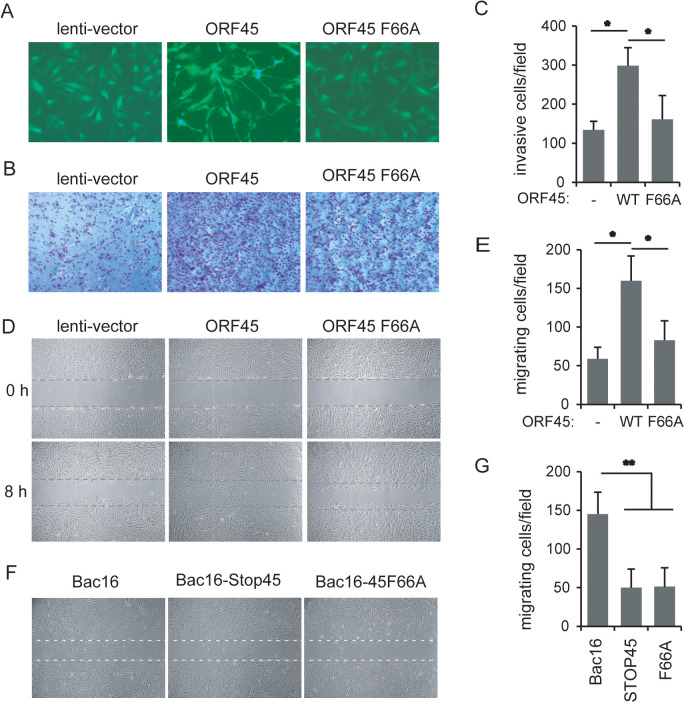
ORF45 promotes cell migration during early primary infection. A-C. HUVECs were transduced with IRES-zsGreen empty vector, ORF45 WT- or F66A-expressing lentiviruses (MOI = 2). Forty-eight hours later, the cells were observed and recorded under fluorescence microscopy (A). A total of 5 × 10^4^ cells/well were subjected to a transwell migration assay with DMEM containing 20% FBS. Cells were left for migration for 24 h and then fixed, stained with crystal violet and recorded by microscopy (B). Migrated cell numbers in each field were counted, and the means were analyzed and are shown (C). D-E. HUVECs were infected with empty or ORF45- or F66A-expressing lentiviruses (MOI = 5). Twelve hours post infection, the cells were subjected to wound healing assays. Representative images are shown (D), and the cells that migrated into the closure per field were counted. The means were analyzed and are shown (E). F. HUVECs were infected with purified Bac16, STOP45 or ORF45F66A recombinant viruses (MOI = 10). Sixty hours after infection, the cells were subjected to a wound healing scratch assay, and representative images are shown. The means of migrated cell numbers were analyzed and are shown (G).

The cells undergoing lytic replication gradually became round and detached while latent infected cells remained attached and spread (Fig 2C), and Filamin A phosphorylation was present in iSLK.219 cells undergoing KSHV lytic replication with ORF45 expression (Fig 1C). Therefore, the Bac16-harboring iSLK-Bac16 cells under lytic induction became detached and stacked at the late stage (Fig 2D). Unlike the wild-type Bac16-infected iSLK cells, the Bac16-STOP45-infected cells did not detach during lytic infection, while reintroduction of ORF45 expression to the ORF45-null Bac16-infected cells restored the detaching phenotype whereas ORF45-F66A reintroduction did not (Fig 2D). These results indicate that ORF45 contributes to cell detachment during lytic replication in adherent cells and may also cause an anchoring-independent cell phenotype during the late lytic cycle. To investigate whether cell motility was affected by ORF45, we introduced ectopic ORF45 or ORF45-F66A into HUVECs using lentiviruses. ORF45 overexpression changed the morphology, whereas this alteration was not observed when ORF45-F66A was overexpressed (Fig 3A). The migration of ORF45-expressing HUVECs but not ORF45-F66A-expressing cells was increased in the transwell cassette (Fig 3B-3C). Similarly, as assessed using the wound healing assay, the cell migration was increased by ORF45 expression but not by ORF45-F66A expression (Fig 3D-3E). When the cells were treated with the RSK inhibitor BI-D1870 or SL0101, the increased ability of ORF45-expressing cells to migrate was greatly attenuated (S2A Fig). These results indicate that ORF45 promotes cell migration in an RSK-dependent manner.

To further investigate the effect of ORF45 on cell motility during lytic cycle, we also performed wound-healing assays of the Bac16-infected cells undergoing lytic replication with different ORF45 expression levels. Within 48 h of primary infection, the cells remained attached and spread, and the wild-type Bac16-harboring cells exhibited a faster wound-healing ability; however, a slower ability was observed in the Bac16-STOP45-harboring cells, similar to the Bac16-ORF45F66A-harboring cells (Fig 3F-3G). Upon additional treatment with BI-D1870 or SL0101 for 24 h, the faster wound-healing ability of Bac16-infected cells undergoing lytic replication was halted whereas the slower wound-healing ability of Bac16-STOP45-harboring cells under conditions of lytic induction was not affected (S2B Fig). Similarly, BI-D1870 or SL0101 treatment reduced the wound-healing ability of cells infected with Bac16 viruses to the levels comparable to those of cells infected with STOP45 viruses, which were minimally affected by these RSK inhibitors (S2C Fig). These results suggest that ORF45, KSHV primary infection and lytic reactivation promote cell migration through RSK activation during the early lytic cycle.

### Filamin A phosphorylation is required for ORF45-induced cell detachment and migration

**Fig 4:**
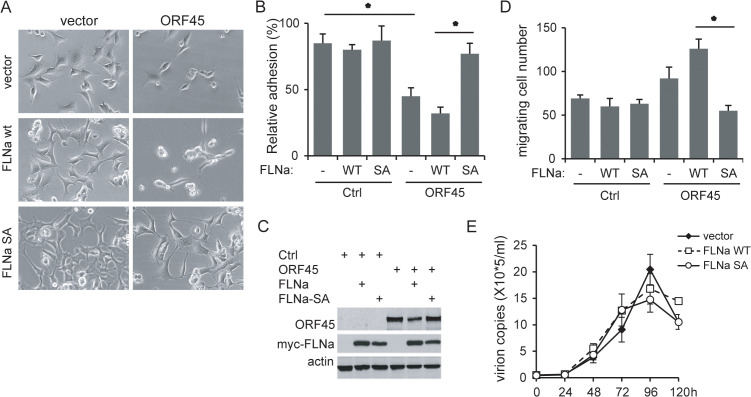
Filamin A phosphorylation is essential for ORF45-induced detachment and migration but not for virion production. A-D. HEK293 cells were transfected with Filamin A WT or S2152A mutated constructs and then infected with empty vector or ORF45-expressing lentiviruses at 8 h after transfection. Twenty-four hours later, the cell morphology was observed by inverted microscopy, and representative images are shown (A). Thirty-six hours after infection, the cells were subjected to analysis for relative adhesion (B), gene expression by western blots (C), and cell migration in the wound healing scratch assay (D). E. Bac16-harboring 239T cells in 6-well plates were transfected with 1.6 μg of Filamin A WT or S2152A construct for 24 h, and then, the cells were induced with 20 ng/ml TPA and 0.3 mm NaB for lytic replication. The supernatants were collected at different time points, and then, the yield of progeny viruses was detected with quantitative PCR analysis.

To further investigate the role of Filamin A phosphorylation in the ORF45-mediated cell motility, we mutated Serine 2152 to Alanine in Filamin A as the phosphorylation-deficient Filamin A construct. Neither wild-type nor mutated Filamin A-S2152A alone affected cell adhesion under normal culture conditions, even though they may alter cell morphology (Fig 4A, left). ORF45 overexpression dramatically decreased cell spread and adhesion, and wild-type Filamin A overexpression did not affect this reduction; however, Filamin A-S2152A overexpression strongly recovered the spread and adhesion of the ORF45-expressing cells (Fig 4A, right). Cell attachment was measured, and neither Filamin A WT alone nor Filamin A-S2152A alone decreased cell adhesion in the absence of ORF45 expression. ORF45 caused a reduction in cell attachment, and Filamin A overexpression augmented it, while Filamin A-S2152A overexpression abolished the reduction in the presence of ORF45 expression (Fig 4B-4C). These results suggest that Filamin A phosphorylation is required for ORF45-induced detachment.

Furthermore, these cells were seeded into transwell cassettes to detect migration across the membranes. Neither Filamin A wild type alone nor Filamin A-S2152A alone affected migration, while ORF45 expression enhanced migration, and Filamin A with ORF45 overexpression augmented migration; however, Filamin A-S2152A overexpression similarly abolished the increased migration (Fig 4D), indicating that ORF45-induced migration also requires Filamin A phosphorylation. However, Filamin A and Filamin-S2152A overexpression did not decrease virion production (Fig 4E). These results suggest that ORF45-induced Filamin A phosphorylation is required for the increased motility of cells undergoing lytic replication but not for viral lytic replication.

### ORF45-Filamin A phosphorylation promotes KSHV de novo infection and cell-to-cell viral transmission

**Fig 5:**
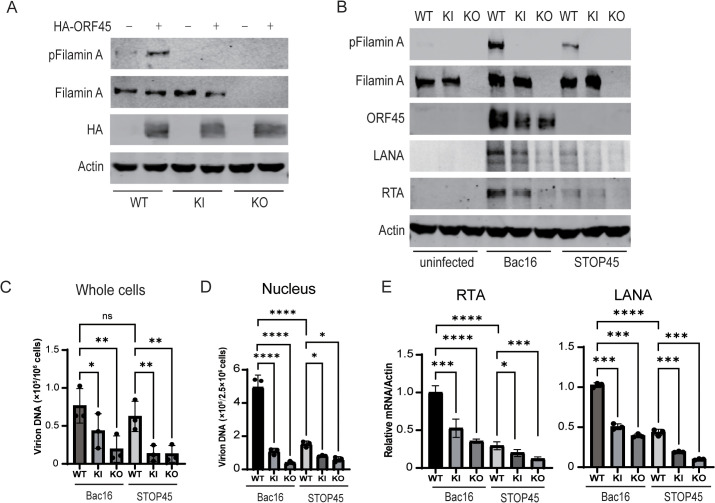
Filamin A KO and S2152A KI reduce KSHV de novo infection of cell-free virions. A-B. Filamin WT, S2152A KI or KO HEK293-mCherry cells were transfected with empty vector or ORF45 expressing plasmid for 24 h (A), or left uninfected or infected with Bac16 or STOP45 viral stocks (MOI = 10) for 24 h (B). The cells were collected and whole cell extracted were subjected to Western blotting analysis as indicated. C-E. Filamin WT, S2152A KI or KO HEK293-mCherry cells were left uninfected or infected with iSLK.Bac16 or iSLK.STOP45 viral stocks (MOI = 10). (C) Two hours post infection, the cells were collected by trypsin digestion, and the intracellular viral DNA and cellular DNA genomes were extracted. (D) Eight hours post infection, the cells were collected and nuclear fractions were isolated, and then viral DNA and cellular DNA genome were extracted from nuclear fractions. The viral DNA copy numbers were detected by real-time PCR and GAPDH DNA was used as internal control. (E) Twenty four hours post infection, the total RNA were extracted, reverse-transcribed and detected by real-time PCR. The levels of viral mRNA were normalized to β-actin mRNA.. *, p < 0.05; **, p < 0.01; ***, p < 0.001; ****, p < 0.0001, t test.

To further investigate the function of Filamin A phosphorylation during KSHV infection, the stable cells lines with Filamin A knockout (KO) or S2152A knockin (KI) were established in HEK293-mCherry cells using CRISPR-Cas9 based gene editing technology (S3 Fig). When ORF45 was overexpressed in these cells, Filamin A phosphorylation was observed in WT cells but not in Filamin A KO or KI cells (Fig 5A). After these cells were infected with Bac16 or STOP45 viruses for 24 h, the primary infection of Bac16 viruses greatly induced Filamin A phosphorylation while STOP45 viral infection weakly increased it in WT cells, and no Filamin A phosphorylation was observed in either Filamin A KO or KI cells (Fig 5B). Stable Bac16 or STOP45 harboring WT, KO or KI cells were further established and then treated with TPA + NaB for 48 h to induce lytic reactivation. High level of Filamin A phosphorylation was observed in Bac16 harboring WT cells and low level was detected in STOP45 harboring cells, while no Filamin A phosphorylation was detected in Filamin A KO or KI cells (S4A Fig). These results validate the Filamin A KO or S2152A KI and the importance of ORF45 in Filamin A phosphorylation during KSHV primary infection and lytic reactivation.

To investigate the role of Filamin A expression and phosphorylation in KSHV primary infection and lytic replication, viral gene expression in Filamin A WT, KI and KO cells with KSHV de novo infection or cells undergoing lytic reactivation was further detected. In cells infected with Bac16 or STOP45 viruses for 24 h, decreased levels of both the lytic gene RTA and the latent gene LANA were observed in Filamin A KI and KO cells compared with those in WT cells infected with Bac16 viruses (Fig 5B). In addition, the lower RTA and LANA levels observed in cells infected with STOP45 viruses were also reduced by Filamin A KI or KO (Fig 5B). However, when stable Bac16- or STOP45-harboring WT, KO or KI cells were induced with TPA + NaB for 48 h, the RTA expression levels and virion yield were lower in STOP45-harboring cells than in Bac16-harboring cells, however, equal levels of RTA and LANA expression were observed in Filamin A WT, KI and KO cells harboring the same viruses (S4A Fig), resulting in equal virion yields at 96 h post-induction (S4B Fig). These results suggest that Filamin A expression and phosphorylation are important for KSHV de novo infection but not for lytic reactivation.

To characterize the key steps by which Filamin A expression and phosphorylation are required for KSHV primary infection, we further investigated the ability of viral entry into cells and nuclei after primary infection in Filamin A WT, KO and KI cells. When these cells were infected with cell-free Bac16 or STOP45 viral stocks (MOI = 10), lower levels of intracellular viral DNA at 2 h post infection were observed in Filamin A KO and KI cells compared with WT cells, whereas the levels were minimally affected between the Bac16-infected cells and the STOP45-infected cells (Fig 5C). These results suggest that Filamin A expression and phosphorylation are important for viral entry into cells, whereas ORF45 is not required at this stage. When the nuclear fractions were isolated at 8 h post infection, the viral DNA copy number was significantly greater in nuclear fractions from Bac16-infected cells compared with that from STOP45-infected cells, and the viral DNA copy numbers of both viruses were further reduced in nuclear fractions from Filamin A KI or KO cells compared with those from WT cells (Fig 5D). These results suggest that both ORF45 and Filamin A phosphorylation are required for intracellular capsid transport to the nucleus. As a result, compared with those in WT cells, the levels of viral gene expression (both the lytic gene RTA and the latent gene LANA) at 24 h post infection were decreased in Filamin A KO and KI cells (Fig 5E). These results suggest that ORF45 and Filamin A phosphorylation are important for KSHV primary infection at early stages and that viral particle-induced Filamin A phosphorylation is likely required for receptor-mediated endocytosis of virions and that ORF45-mediated Filamin A phosphorylation after infection promotes intracellular capsid transport to the nucleus.

**Fig 6:**
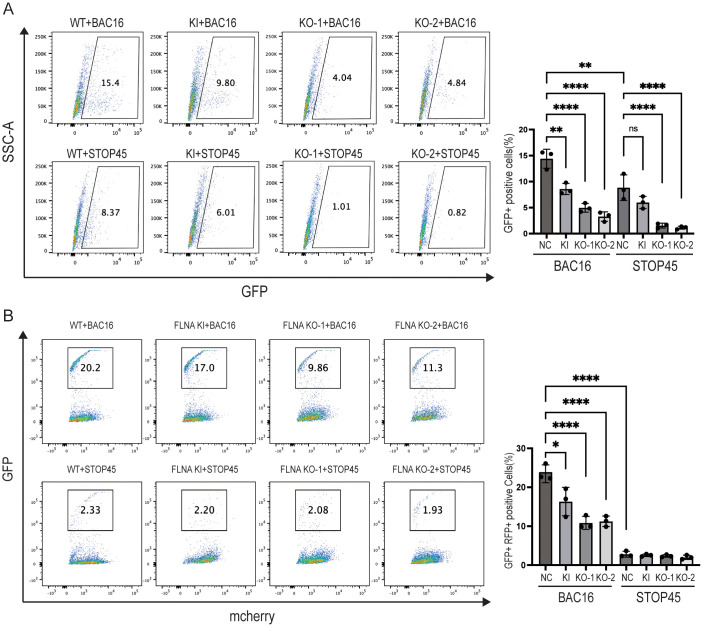
Filamin A KO and S2152A KI reduce KSHV de novo infection and cell-contact mediated viral infection. A. Filamin WT, S2152A KI or KO HEK293-mCherry cells were infected with purified iSLK.Bac16 or iSLK.STOP45 viral stocks (MOI = 10) for 24 h. The cells were harvested and analyzed by FACS flow cytometer. The representative images and the percentages of KSHV-infected HEK293-mCherry cells were calculated and shown in three independent experiments. B. After lytic induction for 72 h, iSLK.Bac16 or iSLK.STOP45 cells were collected and washed twice, and directly added to the monolayer of Filamin WT, S2152A KI or KO HEK293-mCherry cells and co-cultured for additional 24 h. Total cells were harvested and analyzed by FACS flow cytometer. The representative images of mCherry-positive cells are shown and the percentages of KSHV-infected HEK293-mCherry cells were calculated in three independent experiments and shown. **, p < 0.01; ***, p < 0.001; ****, p < 0.0001, t test.

Further, KSHV de novo infection using purified Bac16 or STOP45 virion particles was quantitated in Filamin A WT, S2152A KI or KO HEK293-mCherry cells. The results show that Filamin A KO and S2152A KI similarly decreased the percentage of GFP-positive Bac16-infected cells, a lower percentage of STOP45-infected cells was observed in WT cells and Filamin A KO or S2152A KI additionally dropped the percentages (Fig 6A). Thus, we conclude that ORF45 and Filamin A phosphorylation play the important roles for KSHV de novo infection with cell-free viral particles.

Studies have revealed that cell-contact mediated viral transmission is much more effective than cell-free viral particle transmission for secondary infection [9,10]. The efficiency of cell-to-cell viral infection was further measured using Filamin A WT, KO or S2152A KI HEK293-mCherry cells as recipients, the percentage of GFP-positive KSHV infection from Bac16-infected cells undergoing lytic replication was moderately decreased in FilaminA KI cells and dramatically decreased in KO cells compared with that in WT cells (Fig 6B, top panel), and the infection from STOP45-harboring cells undergoing lytic replication became much lower and was barely affected in all cells (Fig 6B, bottom panel), probably because of the lower cell motility and less virion production. However, when these cells undergoing KSHV reactivation were used as donor cells with normal HEK293 cells as recipients, the percentage of KSHV de novo infection in HEK293 cells was minimally affected (S5 Fig), because the viral gene expression and virion production were not affected by Filamin A KI or KO in recipient cells (S4B Fig). These findings suggest that both ORF45 and Filamin A phosphorylation are required for cell-contact dependent viral infection in recipient cells.

### ORF45-Filamin A phosphorylation promotes cell migration during KSHV primary infection and lytic reactivation at early stage

**Fig 7:**
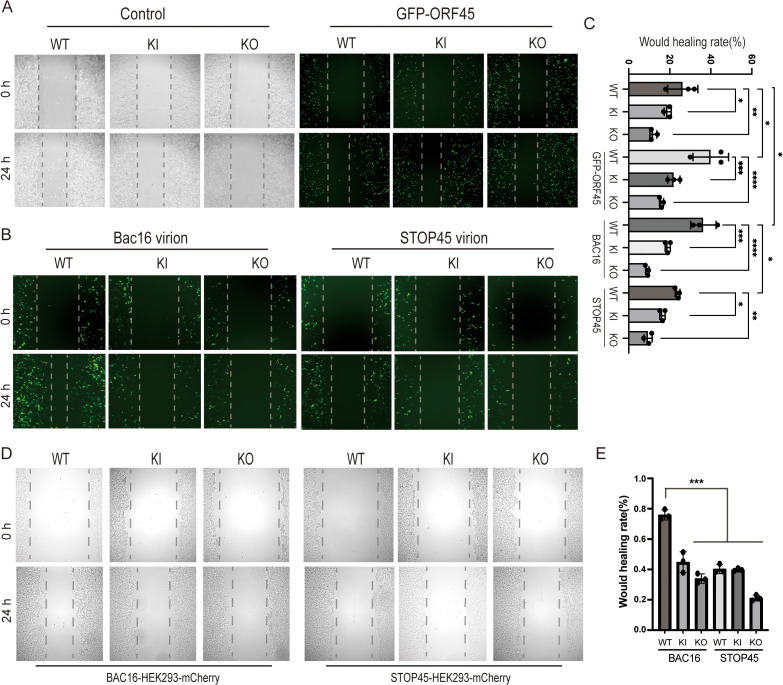
Filamin A KO and S2152A KI reduce cell migration under ORF45 overexpression or during KSHV de novo infection or lytic reactivation. A-C. Filamin WT, S2152A KI or KO HEK293-mCherry cells were transfected with empty vector or ORF45 expressing plasmid for 24 h (A), or left uninfected or infected with iSLK.Bac16 or iSLK.STOP45 viral stocks (MOI = 10) for 24 h (B). The cells were subjected to a wound healing scratch assay, and representative images are shown. (C) The relative wound healing abilities were calculated using ImageJ software and shown. *, p < 0.05; **, p < 0.01; ***, p < 0.001; ****, p < 0.0001, t test. D-E. Bac16 or STOP45-harboring Filamin WT, S2152A KI or KO HEK293-mCherry cells were induced by TPA + NaB for 48 h, and then subjected to a wound healing scratch assay and representative images are shown (D). The relative wound healing abilities were calculated and shown (E). *, p < 0.05; **, p < 0.01; ***, p < 0.001; ****, p < 0.0001, t test.

To confirm the important function of Filamin A expression and phosphorylation in cell mobility during KSHV primary infection and lytic reactivation, the wound healing assays were performed using Filamin A WT, KI or KO cells. The results revealed that both Filamin A KO and KI reduced cell migration, ORF45 overexpression increased cell migration in Filamin A WT cells, whereas the increased migration in presence of ORF45 overexpression was abolished in Filamin A KO and KI cells (Fig 7A and 7C). Similarly, the primary infection of Bac16 viruses promoted the cell migration in WT cells but not in Filamin A KO or KI cells, and the primary infection of STOP45 viruses barely affected the cell migration in all cells (Fig 7B and 7C). After the cells were treated with TPA + NaB for 48 h to induce lytic reactivation, the wound-healing ability was dramatically reduced in the stable Bac16-harboring Filamin A KO or KI cells compared with stable Bac16-harboring WT cells (Fig 7D-7E). In contrast, the wound-healing ability of stable STOP45- harboring cells was much lower and similarly reduced in Filamin A KO or KI cells compared with that in WT cells (Fig 7D-7E). These results suggest that early KSHV primary infection and lytic reactivation at early stage promote cell migration through ORF45-induced Filamin A phosphorylation.

In conclusion, ORF45 induces Filamin A phosphorylation during KSHV primary infection and lytic replication, and then ORF45-Filamin A axis promotes cell detachment and migration and de novo and cell-contact dependent viral infection, to mediate the effective cell movement and viral transmission during lytic cycle, providing the importance for KSHV persistent infection and tumorigenesis.

## Discussion

We observed that the adherent cells undergoing KSHV lytic replication gradually become round and detached during the lytic cycle; however, how and why this phenotype occurs for KSHV infection and diseases remain unknown. In the present study, we demonstrated that ORF45 induces Filamin A phosphorylation through RSK activation during the lytic cycle, and then, the cells undergoing lytic replication become round and detached; consequently, ORF45-expressing and Filamin A-phosphorylated cells exhibit increased cell migration and anchoring independence. Therefore, ORF45-mediated Filamin A phosphorylation promotes KSHV de novo infection and cell-contact mediated viral infection. Then, KSHV-infected adherent cells undergoing primary infection or lytic replication exhibit high motility at the early stage and carry viruses to migrate together for secondary infection and spread. These results revealed that ORF45 induces cell detachment, migration and invasion of KSHV-infected cells during lytic replication and promotes KSHV de novo infection and cell-contact dependent viral infection by inducing Filamin A phosphorylation, indicating that ORF45-mediated Filamin A phosphorylation plays an essential role in KSHV viral transmission and pathogenesis.

Our results show that both ORF45 and Filamin A phosphorylation are important of both KSHV de novo infection of cell-free virion particles and cell-contact viral infection from cells undergoing lytic replication. Although the ORF45 protein cannot trigger RSK activation and Filamin A phosphorylation on cell surfaces, it can be released as a tegument protein from virion particle to the cytoplasmic compartment quickly after virions enter cells through receptor-mediated endocytosis. In addition, it is expressed immediately after primary infection, subsequently inducing RSK activation and Filamin A phosphorylation during the immediately early stage of primary infection. Consequently, the actin cytoskeleton will be reprogrammed to promote the movement of capsids to nuclei. Therefore, ORF45-induced Filamin A phosphorylation starts from the immediately early stage of primary infection and promotes the de novo infection, likely facilitating the capsid transport to the nucleus.

In addition to B cells, KSHV-infected human blood endothelial cells (BECs), lymphatic endothelial cells (LECs) and oral keratinocytes in vivo [6,7] support the natural KSHV lytic cycle to provide donors and virion reservoirs for viral transmission through both cell-associated and cell-free viral particles, respectively. However, the yield and infectious efficiency of cell-free viral particles are poor; thus, cell-contact viral infection may represent the main mode of viral transmission under natural conditions [9,10]. Thereafter, the release and movement of virus-replicating cells is a determinant for this kind of viral transmission; these cells either migrate and invade local lesions or become circulating endothelial cells, and then carry and transmit cell-associated viral particles through adhesion and cell contact.

The latently infected endothelial and epithelial cells show adhesive phenotypes, while these cells become detached at the late lytic stage (Fig 2). Although several KSHV gene products regulate cell migration for angiogenesis and tumorigenesis [44–49], only viral thymidine kinase (TK), by acting as a tyrosine kinase, has been reported to disrupt adhesion through FAK, paxillin and RhoA-ROCK-myosin II signaling [50,51]. In the present study, the sustained ORF45 expression and RSK activation induced Filamin A Ser2152 phosphorylation and then promoted the detachment and migration of adherent cells undergoing lytic replication, while ORF45-null cells undergoing lytic replication lost Filamin A phosphorylation and maintained focal adhesion. The increased cell detachment and movement enables cell migration during the early lytic cycle and the spread of virus-replicating cells during the late lytic stage to facilitate cell-contact mediated viral transmission. However, TK might not be the main modulator for detachment of cells under lytic replication because TK expression was not strongly affected by ORF45 loss during lytic replication in the Bac16-STOP45-infected cells compared with the Bac16 wild-type-infected cells [14]; thus, ORF45-mediated signaling would play an important role in this process.

Although Filamin A phosphorylation induced by ORF45 regulates cell detachment and motility, it is not essential for viral lytic replication (Figs 4E and S4). First, KSHV-positive BCBL1 and BC1 cells do not express detectable Filamin A (S6 Fig), but they support effective lytic replication and produce the progeny virions, indicating that KSHV lytic replication does not absolutely require Filamin A expression and phosphorylation. Second, Filamin A phosphorylation is induced by cytoplasm-localized ORF45, while nucleus-restricted ORF45 loses the induction of Filamin A phosphorylation (Fig 1D), opposite to the essential nuclear localization of ORF45 in lytic replication [41]. Thus, we can conclude that Filamin A phosphorylation induced by ORF45-RSK signaling plays critical roles in cell motility and viral transmission rather than lytic replication.

The viral-encoded antiapoptotic proteins are upregulated during the lytic stages and then protect cell survival by inhibiting anoikis [52,53]. Filamin A phosphorylation is associated with ORF45 expression in cells undergoing KSHV lytic replication, and these cells show loss of spreading and focal adhesions, indicating anchoring independence in late KSHV lytic replication. Since the cells eventually produce virion particles, the exfoliated cells can migrate along the layer of endothelial and epithelial cells or become circulating cells, and then, virus-replicating cells will carry cell-associated viral particles and transmit viral infection over a long distance by cell contact and re-attachment, representing an effective mode of viral transmission in vivo.

In addition, ORF45 expression during KSHV primary infection might promote cell migration and invasion, suggesting that KSHV-infected endothelial and epithelial cells exhibit increased migration and invasiveness during primary infection. Since continued lytic replication and reinfection are essential for KSHV tumorigenesis [5] and a cluster of lytic genes, including ORF45, are expressed in early primary infection [54], the ORF45-induced effects on cell morphology and motility would play an important role in tumorigenesis and progression of KSHV-related diseases.

In conclusion, we characterized Filamin A as a novel substrate of ORF45-mediated RSK activation and revealed its important function in altering the cell morphology and motility of adherent cells undergoing KSHV primary infection and lytic replication and then promoting de novo infection and cell-contact dependent viral infection. We then elucidated one mechanism by which KSHV lytic replication induces cell detachment and movement and promotes the infection of cell-free and cell-associated viral particles for viral transmission, persistent infection and pathogenesis.

## Materials and methods

### Cell lines, reagents and plasmids

SLK cells, HEK293 cells and HEK293T cells were maintained in Dulbecco’s modified Eagle’s medium supplemented with 10% fetal bovine serum (FBS), 1% L-glutamine and penicillin-streptomycin. Wild type iSLK.Bac16, ORF45-null iSLK.STOP45 and ORF45 F66A mutated iSLK.ORF45F66A cells [14], nucleus-restricting (RN) and cytoplasm-residing (RC) ORF45-expressing iSLK.Bac16 cells [41], KSHV-positive iSLK.219 cells [55], were described previously and were maintained in DMEM supplemented with 10% FBS, glutamine, and antibiotics including G418, hygromycin or puromycin. Human umbilical vein epithelial cells (HUVECs) were purchased and cultured in complete endothelial cell medium supplemented with growth factors (ScienCell, Shanghai, China). Hygromycin B, puromycin and fibronectin were obtained from Invitrogen (Carlsbad, CA). G418, 12-O-tetradecanoylphorbol-13-acetate (TPA), sodium butyrate (NaB), and doxycycline were purchased from Sigma (St. Louis, MO). pcDNA3-Filamin A (FLNA) WT- and S2152A-expressing plasmids were from Addgene. pKH3, pKH3-RSK2, RSK2 Y707A (constitutive active), and RSK2 K100A/Y707A (kinase dead) were described previously [17,18]. ORF45 mutated constructs and the recombinant viral strains were described previously [14,41]. Transfection was performed with Lipofectamine 2000 (Invitrogen) according to the manufacturer’s instructions. RSK inhibitors BI-D1870 and SL0101 were purchased from MedChemExpress.

### Antibodies

Rabbit phospho-specific antibodies against FLNA (Ser-2152) and total FLNA were purchased from Cell Signaling Technology (Beverly, MA). Immunohistochemical phosphorylated FLNA Ser2152 antibody was obtained from Genetex, Inc. (Irvine, CA). The rat anti-LANA antibody was obtained from Advanced Biotechnologies, Inc. (Columbia, MD). Mouse anti-RTA and anti-ORF45 antibodies were described previously [26,41]. Immunoblotting analysis was performed with species-matched secondary antibodies labeled with IRDye680 or IRDye800 and visualized with the Licor Odyssey system.

### Lentivirus preparation and transduction

Recombinant lentiviral stocks were prepared in 293T cells through a triple plasmid co-transfection procedure with 1 mg/ml polyethyleneimine (PEI, Polysciences catalog number 23966). Briefly, 293T cells were seeded on 10 cm dishes, and cells at 60% confluence were used for transfection. A total of 20 μg plasmid with equal amounts of lentiviral plasmid and the package plasmids pSPAX2 and pMD2G was mixed with 66.7 μL of PEI in Opti-MEM medium. The mixture was added to the cells and incubated for 8 h. The lentivirus-containing supernatants were collected at 48 h post-transfection.

HUVECs or SLK cells were consequently transduced with lentiviruses following the standard procedure. For construction of Tet-on inducible ORF45 cells, full length ORF45 fragment was cloned into the pLVX-tight-puro vector, lentiviruses were made from the pLVX-Tet-on-advanced vector and pLVX-tight-puro-ORF45 plasmids with package plasmids, and the cells were transduced with both lentiviral stocks and selected with G418 or puromycin for 2 weeks.

### Establishment of Filamin A knockout (KO) and S2152A knock-in cell lines

HEK293 cells were transfected with pmCherry-C1 plasmid using Lipofectamine 2000 (11668027, Invitrogen, USA), followed by selection with 1 μg/ml G418 for 14 days. The mCherry-positive cells were isolated based on red fluorescence using a FACSAria III flow cytometer, pooled and passaged as stable HEK293-mCherry cell line.

To generate FLNA knockout (KO) and FLNA S2152A knock-in (KI) cell line, sgRNAs targeting the FLNA CDS or S2152A site were designed using the online tool (https://chopchop.cbu.uib.no/). The corresponding oligonucleotides were annealed and ligated into the BsmBI-digested lentiCRISPR v2 plasmid. The FLNA KO sgRNA-expressing plasmids were transfected into HEK293-mCherry cells using Lipofectamine 2000 for 24 h, followed by 1 μg/ml puromycin selection for 14 days. The single clones were individually picked, expanded and validated by Western Blots with anti-FLNA antibody as stable FLNA KO HEK293-mCherry cell line.

To generate FLNA S2152A KI HEK293-mCherry cell line, a donor plasmid, pcDNA3.1-FLNA S2152A, containing homology arms and the point mutation sequence, was linearized with MluI digestion plus gel purification and then co-transfected with the lentiCRISPR v2 plasmid at equimolar concentrations into HEK293-mCherry cells, along with 10 μmol/L SCR7 (S7742, Selleck Chemicals, USA). Seventy-two hours post-transfection, cells were digested into single-cell suspensions and seeded into 96-well plates. Single-cell clones were isolated, expanded, and validated by Western Blots with anti-FLNA (Ser-2152) specific antibody following 20% FBS stimulation for 0.5 h.

The sequences of sgRNAs are used as below:

FLNA KO-sg-1: CCTACGTTCAGGACCGTGGCGAT;
FLNA KO-sg-2: CCACGGTGATGGCACGCACACCA;
FLNA KO-sg-3: AGTGGAGTACACGCCTTACGAGG;
FLNA KI-sg: CCTTCAGTGGCCAACGTTGGTAG.

To establish stable Bac16 or STOP45-harboring cells, Filamin WT, S2152A KI or KO HEK293-mCherry cells were infected with Bac16 or STOP45 virions (MOI = 10) for 48 h, and then selected with 200 μg/ml hygromycin for an additional 2 weeks. The single colonies were pooled and passaged in the presence of 200 μg/ml hygromycin.

### Immunofluorescence staining

KSHV harboring iSLK-Bac16 cells were allowed to adhere and spread on poly-L-lysine-coated coverslips. At the indicated times, the cells were fixed, permeabilized and stained with rabbit anti-p-Filamin A and mouse anti-ORF45 antibodies and then with donkey secondary antibodies labeled with Alexa 555 or Alexa 647 dye (Invitrogen). Stained cells were mounted and visualized with a Zeiss LSM880 confocal microscope under an oil lens.

### Cell-free and cell contact-mediated viral infection

For cell-free viral infection, iSLK.Bac16 or iSLK.STOP45 cells were induced with 1 μg/ml Dox and 1 mM NaB for 96 h, the supernatants were collected and the viral stocks were prepared and purified with ultracentrifugation (100,000 g) as described previously [18]. Next, the purified virions (MOI = 10) were added to cells in the presence of 8 μg/ml polybrene. After the cells were centrifuged at 700 g for 1 h and incubated for additional 6 h in a 37 °C CO_2_ incubator, the medium was refreshed.

For cell contact-mediated viral infection, iSLK.Bac16 or iSLK.STOP45 cells were induced with 1 μg/ml Dox and 1 mM NaB for 48 h, or Bac16 or STOP45-harboring HEK293-mCherry cells were induced with 20 μg/ml TPA plus 1 mM NaB for 48 h, and then washed twice. Then, the medium was replaced with fresh medium. The detached cells were collected at 72 h and added directly to HEK293-mCherry or HEK293 cells at a 1:1 ratio for cell-mediated viral infection as the donor and recipient cells, respectively.

### Transwell cell migration assay

Eight μm pore size transwell membranes (Costar) were coated with 10 μg/ml fibronectin at 4 °C overnight. For HUVECs, a total of 5 × 10^4^ cells were seeded into the upper chamber in DMEM supplemented with 1% FBS, and the lower chamber was filled with 600 μL of DMEM with 20% FBS. Cells were incubated for an additional 24 h for cell migration. The transwell was then fixed in 75% cold ethanol for 15 min and stained with 0.5% crystal violet for 20 min. Cells in the upper chamber were removed with a cotton swab and washed twice. Then, the migrated cells on the lower side of the membrane were visualized with a Zeiss Axio Observer Z1 inverted microscope.

### Wound healing scratch assay

HUVECs or HEK293 cells were grown on 6-well plates as monolayers. After viral transduction for 12 h, the cells were scratched using a 200-μl pipette tip to obtain wounds of the same width. Then, the cells were washed and allowed to migrate in DMEM for an additional 8–24 h. The number of migrated cells entering the scratched area was recorded and counted under an inverted microscope. Three independent results were analyzed, and the means were calculated.

### Focal adhesion assay

A 96-well plate was coated with 10 μg/ml fibronectin in PBS at 4 °C overnight. The wells were then blocked with 3% BSA in PBS for 2 h at room temperature. Equal numbers of 293 cells in DMEM containing 0.1% FBS were seeded on fibronectin-precoated wells, and the cells were left to adhere for 1 h in a 37 °C CO_2_ incubator. Then, the wells were washed with a low shear washing method to remove nonadherent cells, and the unwashed cells were used as a 100% control for quantification. Adherent cells were rinsed with PBS once, fixed in 4% formaldehyde for 30 min, and stained with 0.5% crystal violet for 40 min. The cells were then washed three times with PBS, solubilized in 100 µl of 10% acetic acid, and the absorbance was measured at 650 nm by a UV-visible spectrophotometer.

### Statistical analysis

Significant differences between experimental groups were determined by two-tailed unpaired Student’s t-test. Values of p < 0.05 were considered statistically significant.

## Supporting information

S1 Fig RSK inhibitors suppress ORF45-induced Filamin A phosphorylation. The solvent DMSO, 10 μM BI-D1870 or 10 μM SL0101 was added to ORF45-overexpressing HEK293 cells (A), iSLK.Bac16 cells undergoing Dox + NaB-induced lytic reactivation (B) and HEK293 cells infected with Bac16 virions (MOI = 10) (C) for 24 h, the cells were collected and the whole cell extracts were subjected to Western Blotting analysis to detect the level of Filamin A phosphorylation.
(TIF)

S2 Fig The treatment of RSK inhibitors attenuates ORF45-increased ability of cell migration. GFP-ORF45-overexpressing HEK293 cells (A), iSLK.Bac16 cells or iSLK.STOP45 cells undergoing Dox + NaB-induced lytic reactivation (B) and HEK293 cells infected with Bac16 or STOP45 virions (MOI = 10) (C) were subjected to a wound healing scratch assay in presence of DMSO, 10 μM BI-D1870 or 10 μM SL0101 treatment. The representative images and the relative wound healing abilities were shown. *, p < 0.05; **, p < 0.01; ***, p < 0.001; ****, p < 0.0001, t test.
(TIF)

S3 Fig Validation of Filamin A KO and S2152A KI stable HEK293-mcherry cells. Stable Filamin A KO and S2152A KI cells were established using CRISPR-Cas9 based gene editing procedure in HEK293-mCherry cells, and the single cell clones were selected, picked and expanded sequentially. A. After the whole cell extracts were prepared, three Filamin A KO cell lines were validated by Western blotting analysis with anti-Flamin A antibody. B. After the cells were serum starved overnight and stimulated with 20% FBS for 30 min, the whole cell extracts were prepared and two Filamin A S2152A KI cell lines were validated by Western blotting analysis with anti-Filamin A (Ser-2152) phosphorylation specific antibody.
(TIF)

S4 Fig Filamin A KO or S2152A KI does not affect KSHV lytic reactivation. A. Stable Bac16 or STOP45-harboring Filamin A WT, KO and S2152A KI HEK293-mCherry cells were treated with TPA + NaB for 72 h, the cells were collected and whole cell extracts were subjected to Western Blotting analysis to detect the viral gene expression. B. After the cells were induced with TPA + NaB for 96 h, the supernatants were collected and viron DNA were extracted and analyzed by real-time PCR. ns, no statistical significance, t test.
(TIF)

S5 Fig Filamin A KO and S2152A KI in donor cells do not affect KSHV cell-to-cell viral transmission. A. Stable Bac16 or STOP45-harboring Filamin WT, S2152A KI or KO HEK293-mCherry cells were induced with TPA + NaB for 72 h, and then cells were collected and washed twice, and directly added to the monolayer of HEK293 cells and co-cultured for additional 24 h. Total cells were harvested and GFP-positive mCherry-negative cells were analyzed by FACS flow cytometer. The representative images and the percentages of KSHV-infected HEK293 cells were calculated in three independent experiments and shown. ns, no statistical significance; **, p < 0.01; t test.
(TIF)

S6 Fig The expression levels of Filamin A in KSHV-positive cells. The whole cell extracts of HEK293, iSLK.Bac16, BCBL1 and BC1 cells were subjected to Western Blotting analysis as indicated to detect the expression of Filamin A.
(TIF)
