# Supplementary figures and images for: ORF45-induced Filamin A phosphorylation promotes cell motility and cell-contact dependent viral infection of Kaposi’s sarcoma-associated herpesvirus
Source: PLoS Pathog. 2025 Nov 24;21(11):e1013737. doi: 10.1371/journal.ppat.1013737 (PMC12677772; doi:10.1371/journal.ppat.1013737)

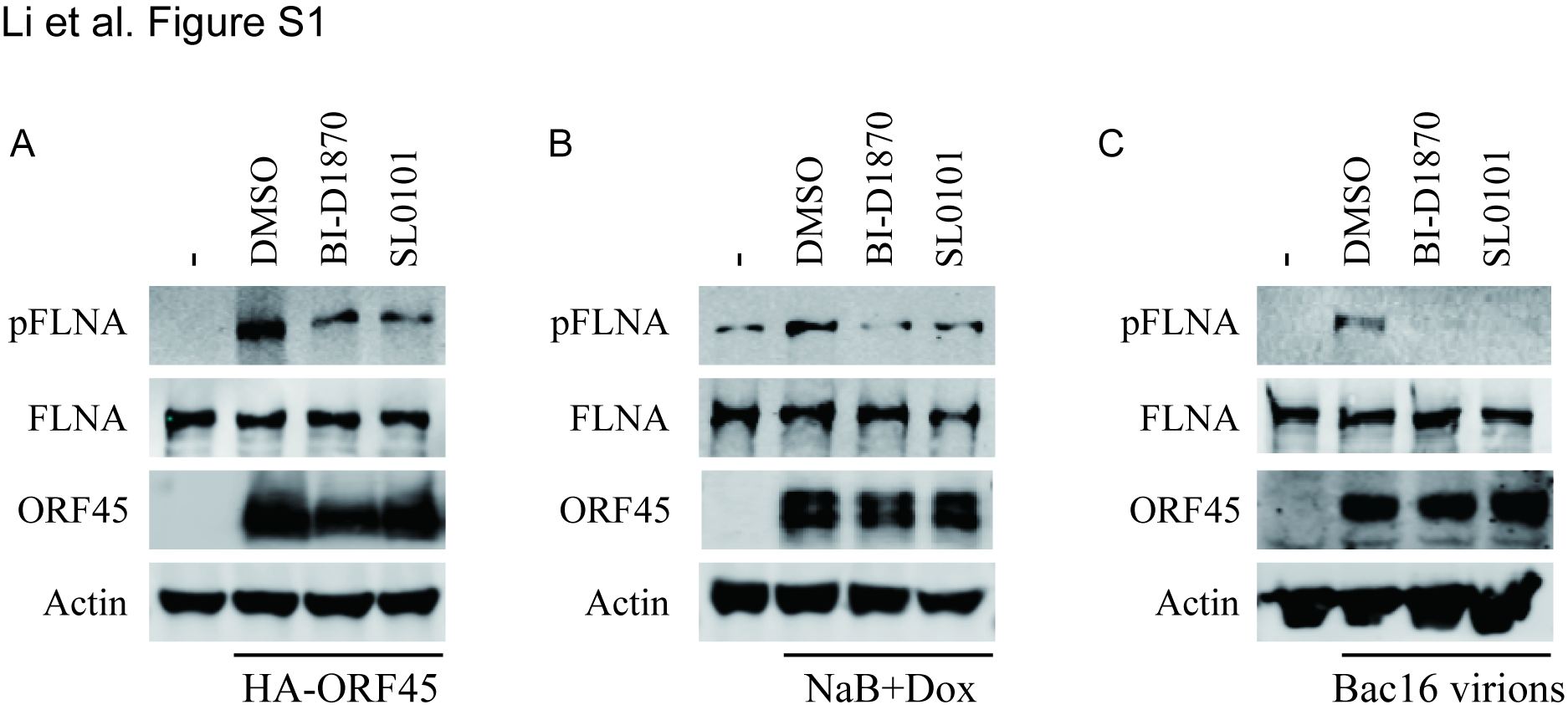

Supplement: S1 Fig — The solvent DMSO, 10 μM BI-D1870 or 10 μM SL0101 was added to ORF45-overexpressing HEK293 cells (A), iSLK.Bac16 cells undergoing Dox + NaB-induced lytic reactivation (B) and HEK293 cells infected with Bac16 virions (MOI = 10) (C) for 24 h, the cells were collected and the whole cell extracts were subjected to Western Blotting analysis to detect the level of Filamin A phosphorylation. (TIF) [file ppat.1013737.s001.tif]

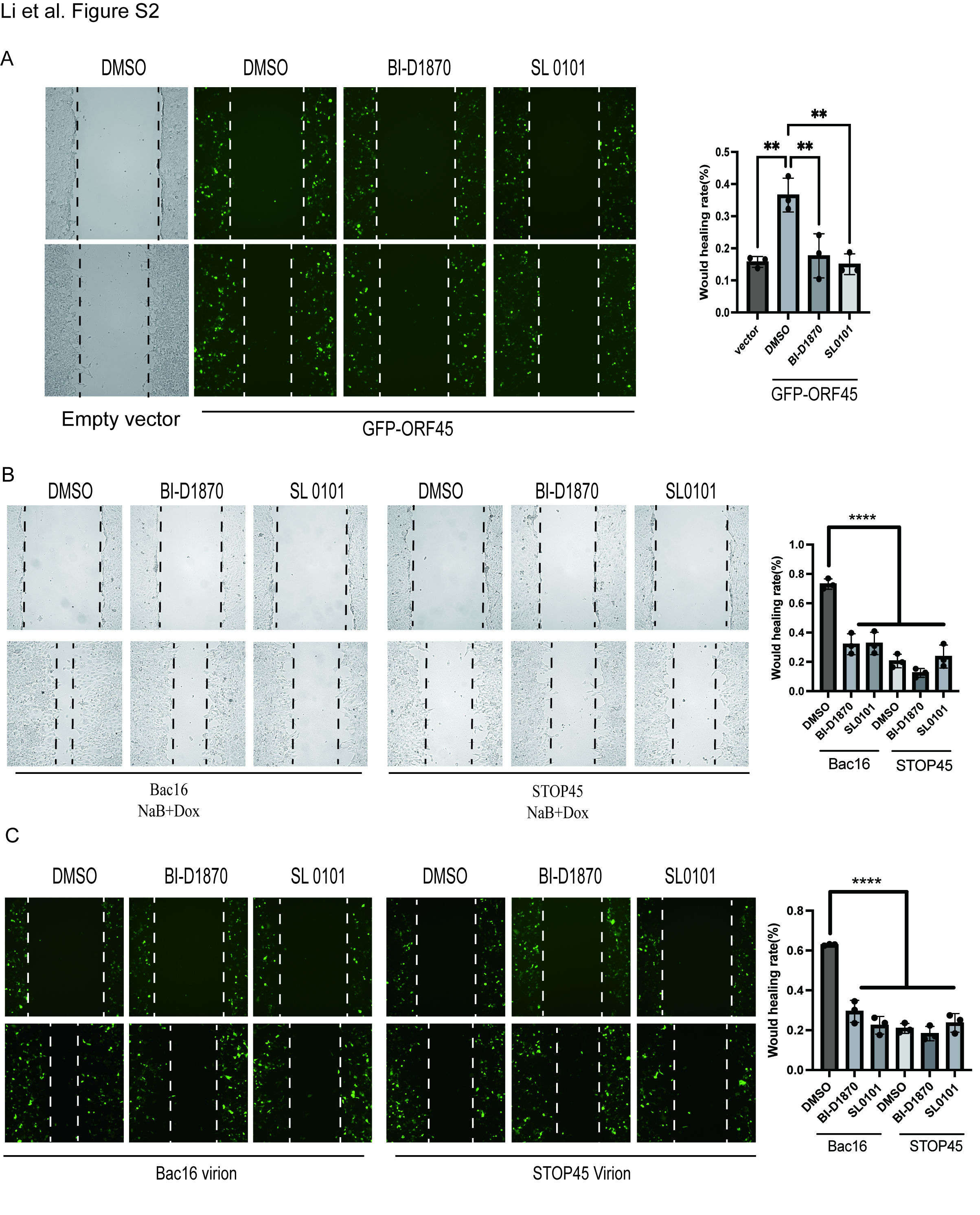

Supplement: S2 Fig — GFP-ORF45-overexpressing HEK293 cells (A), iSLK.Bac16 cells or iSLK.STOP45 cells undergoing Dox + NaB-induced lytic reactivation (B) and HEK293 cells infected with Bac16 or STOP45 virions (MOI = 10) (C) were subjected to a wound healing scratch assay in presence of DMSO, 10 μM BI-D1870 or 10 μM SL0101 treatment. The representative images and the relative wound healing abilities were shown. *, p < 0.05; **, p < 0.01; ***, p < 0.001; ****, p < 0.0001, t test. (TIF) [file ppat.1013737.s002.tif]

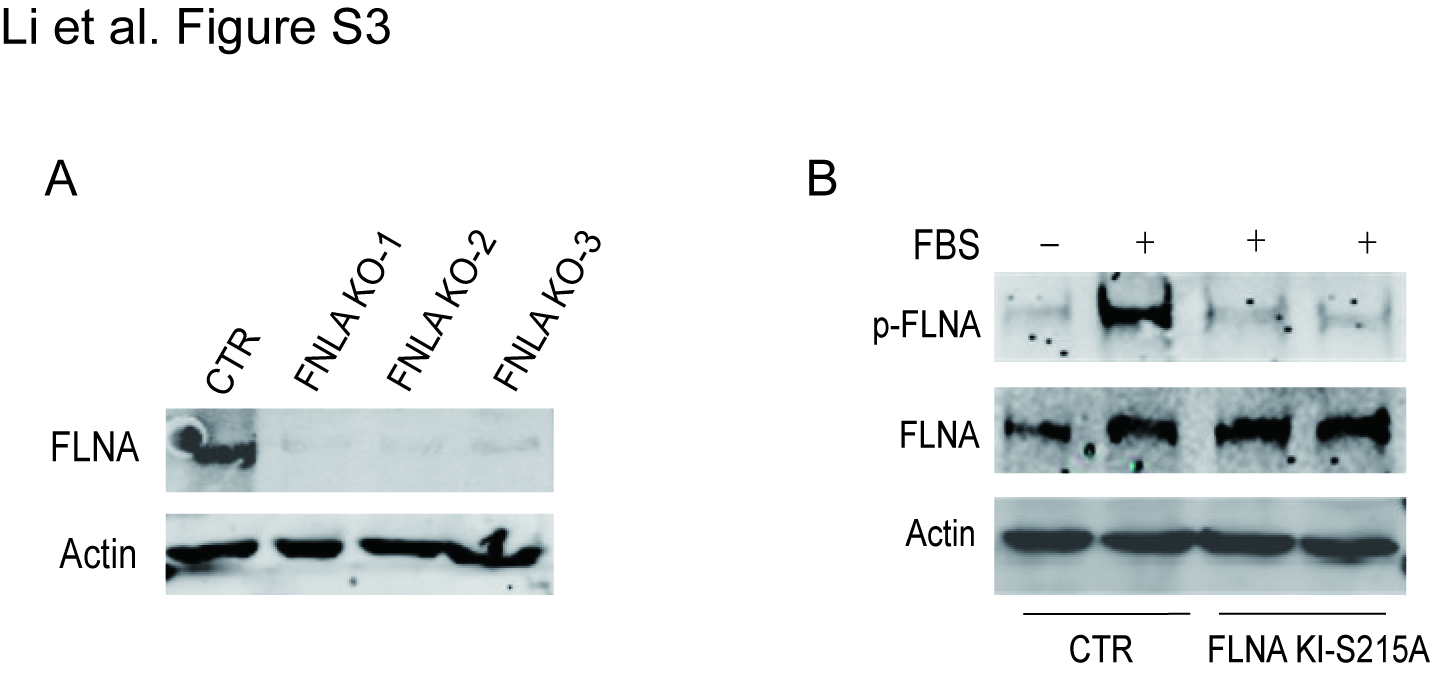

Supplement: S3 Fig — Stable Filamin A KO and S2152A KI cells were established using CRISPR-Cas9 based gene editing procedure in HEK293-mCherry cells, and the single cell clones were selected, picked and expanded sequentially. A. After the whole cell extracts were prepared, three Filamin A KO cell lines were validated by Western blotting analysis with anti-Flamin A antibody. B. After the cells were serum starved overnight and stimulated with 20% FBS for 30 min, the whole cell extracts were prepared and two Filamin A S2152A KI cell lines were validated by Western blotting analysis with anti-Filamin A (Ser-2152) phosphorylation specific antibody. (TIF) [file ppat.1013737.s003.tif]

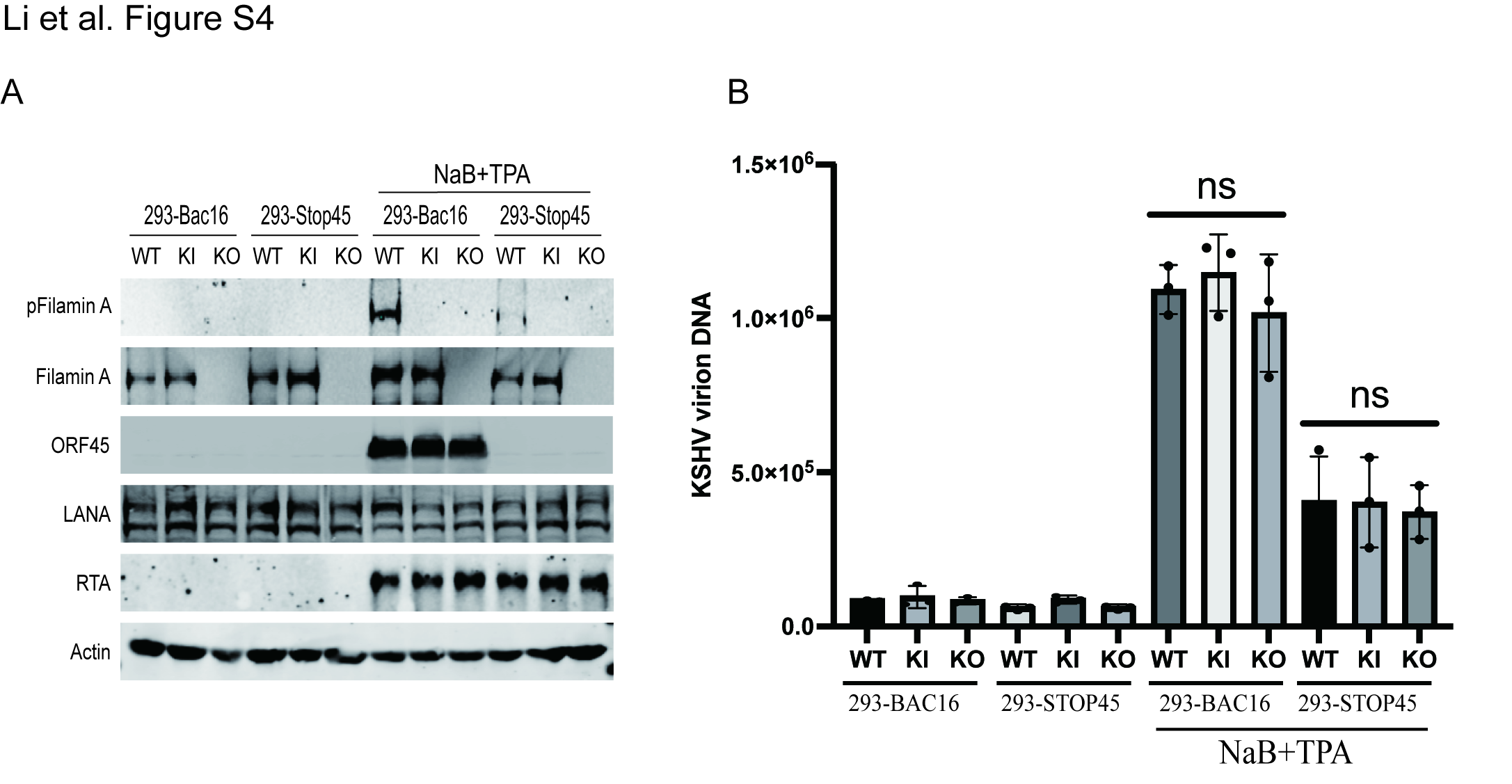

Supplement: S4 Fig — A. Stable Bac16 or STOP45-harboring Filamin A WT, KO and S2152A KI HEK293-mCherry cells were treated with TPA + NaB for 72 h, the cells were collected and whole cell extracts were subjected to Western Blotting analysis to detect the viral gene expression. B. After the cells were induced with TPA + NaB for 96 h, the supernatants were collected and viron DNA were extracted and analyzed by real-time PCR. ns, no statistical significance, t test. (TIF) [file ppat.1013737.s004.tif]

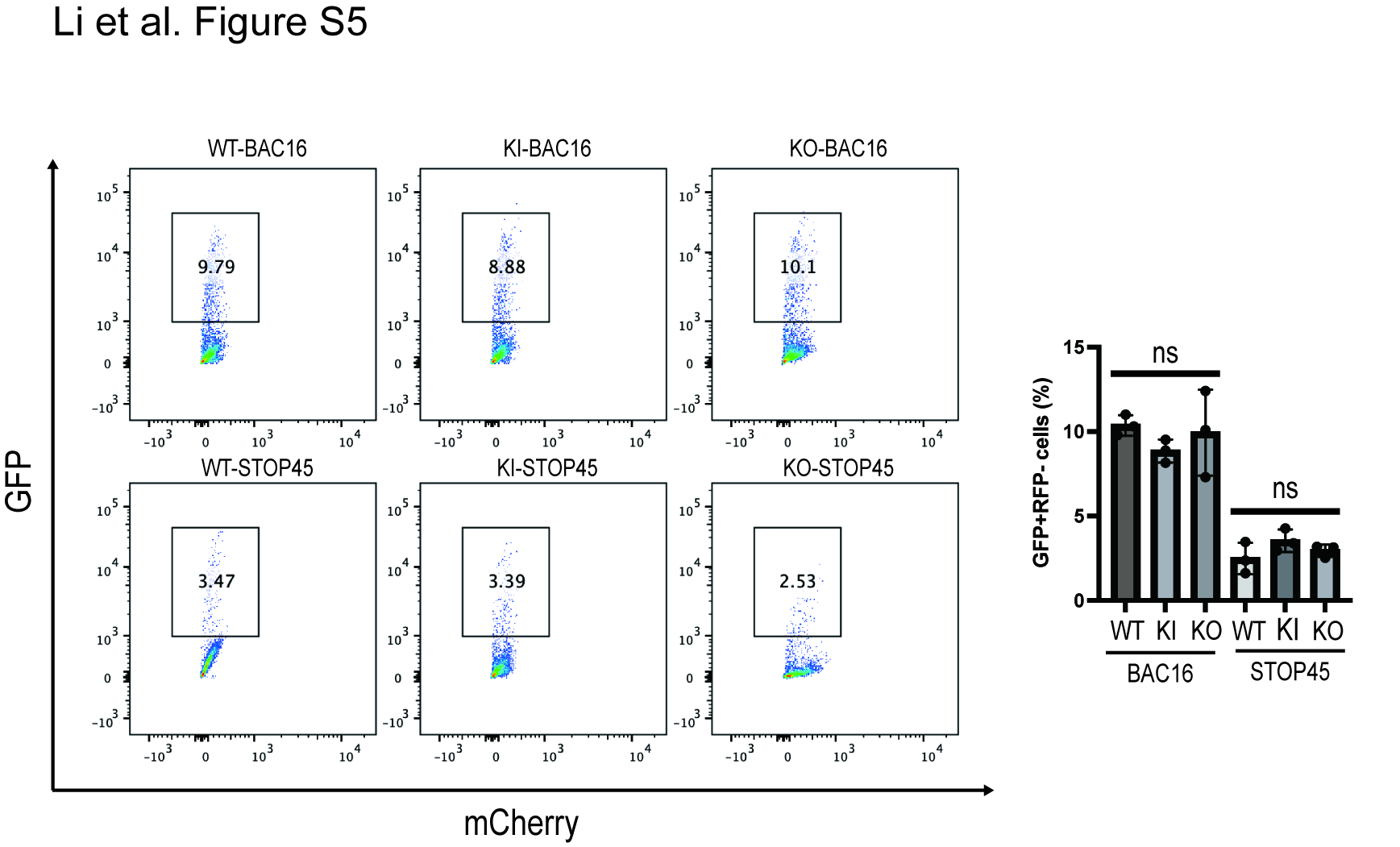

Supplement: S5 Fig — A. Stable Bac16 or STOP45-harboring Filamin WT, S2152A KI or KO HEK293-mCherry cells were induced with TPA + NaB for 72 h, and then cells were collected and washed twice, and directly added to the monolayer of HEK293 cells and co-cultured for additional 24 h. Total cells were harvested and GFP-positive mCherry-negative cells were analyzed by FACS flow cytometer. The representative images and the percentages of KSHV-infected HEK293 cells were calculated in three independent experiments and shown. ns, no statistical significance; **, p < 0.01; t test. (TIF) [file ppat.1013737.s005.tif]

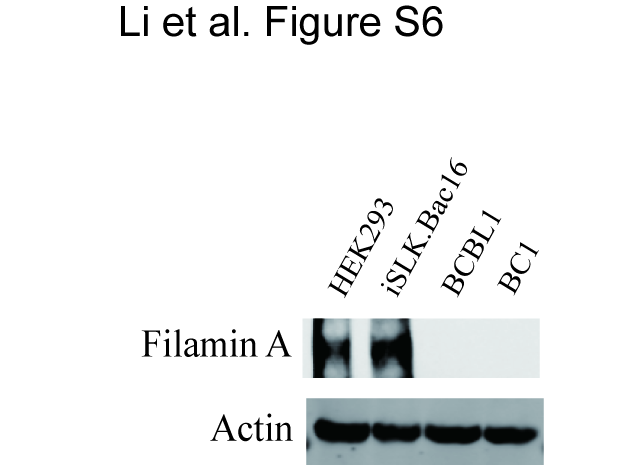

Supplement: S6 Fig — The whole cell extracts of HEK293, iSLK.Bac16, BCBL1 and BC1 cells were subjected to Western Blotting analysis as indicated to detect the expression of Filamin A. (TIF) [file ppat.1013737.s006.tif]
